# Potential of variegated lady beetle *Hippodamia variegata* in management of invasive tomato potato psyllid *Bactericera cockerelli*

**DOI:** 10.1002/ps.7247

**Published:** 2022-11-03

**Authors:** Shovon Chandra Sarkar, Stephen Paul Milroy, Wei Xu

**Affiliations:** ^1^ Food Futures Institute Murdoch University Perth Western Australia Australia

**Keywords:** predation, invasive pest, life table, demographical parameters, biological control

## Abstract

**BACKGROUND:**

The tomato potato psyllid, *Bactericera cockerelli* (Šulc) is a new invasive pest in Western Australia, which may disperse across the whole of Australia within a few years and cause significant economic losses. Chemical control is the most widely used approach to manage *B. cockerelli*, but insect resistance, chemical residue and effects on non‐target species have become an increasing concerned. Therefore, in this study, the biocontrol potential of variegated lady beetle, *Hippodamia variegata* (Goeze) was investigated. The impact of utilizing *B. cockerelli* as a food source on the predator's development and reproduction was assessed by formulating age‐stage, two sex life tables. The predatory potential of *H. variegata* on *B. cockerelli* nymphs was assessed in a closed arena and the effects of releasing *H. variegata* for the control of *B. cockerelli* were then evaluated.

**RESULTS:**

*H. variegata* could successfully develop and oviposit when feeding on *B. cockerelli*. However, both survival and the rate of development were higher for *H. variegata* feeding on *Myzus persicae* (Sulzer) than *B. cockerelli* or a mixed population of *B. cockerelli* and *M. persicae*. A type II functional response was observed for *H. variegata*. In the greenhouse, the releases of *H. variegata* larvae reduced the number of *B. cockerelli* nymphs by up to 66% and adults by up to 59%, which positively influenced the plant chlorophyll content and biomass.

**CONCLUSIONS:**

This study demonstrated the potential of the resident generalist predator, *H. variegata* as a biocontrol agent for the invasive pest, *B. cockerelli*, which may help improving current management strategies. © 2022 The Authors. *Pest Management Science* published by John Wiley & Sons Ltd on behalf of Society of Chemical Industry.

## Graphical abstract

Resident predator *Hippodamia variegata* successfully reproduced and established populations when feeding on invasive pest, *Bactericera cockerelli*. Therefore, *H. variegata* could be a potential biological control agent for *B. cockerelli*. This is the first report of a generalist predator feeding and developing on the invasive *B. cockerelli* in Australia.

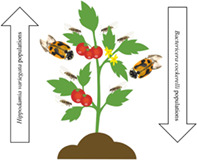

## 1 INTRODUCTION

The tomato potato psyllid, *Bactericera cockerelli* Šulc (Hemiptera: Triozidae), is currently considered one of the most economically important threats to potato and tomato production. While these two crops are its preferred hosts, it has a potential host range exceeding 20 plant families and only within Solanaceae it can develop on 40 species.^1^, ^2^, ^3^, ^4^ *B. cockerelli* is native to southern North America. It was first reported in Australia as an invasive pest species in Perth, WA in February 2017 but was already widespread around the Perth city.^5^ It is believed that suitable climatic conditions, their migratory and overwintering behavior and the availability of numerous crop and non‐crop solanaceous hosts will facilitate *B. cockerelli* to invade most other states of Australia. *B. cockerelli* can cause severe economic loss through either direct or indirect damage.^6^ Directly, it can damage host plants through phloem feeding, and indirectly it acts as a vector of *Candidatus* Liberibacter solanacearum (CLso) (Rhizobiales: Phyllobacteriaceae) (zebra chip), a bacterial pathogen which can reduce crop yields and quality significantly. In extreme cases, total yield loss can occur.^3^, ^7^, ^8^

Control of *B. cockerelli* has mainly relied on insecticides (such as the Diamide, Spinosyns, Carbamate, Neonicotinoid, Spirotetramat and Pyrazole groups) since it has become prominent as an agricultural pest.^6^, ^9^, ^10^, ^11^, ^12^ However, *B. cockerelli* has already shown a capacity to develop resistance.^13^, ^14^, ^15^, ^16^, ^17^ For example, in California, Liu and Trumble,^13^ observed *B. cockerelli* resistance to imidacloprid (based on the LC_50_ for *B. cockerelli* nymphs). Prager *et al*.,^15^ also reported resistance of *B. cockerelli* to imidacloprid in southern Texas and there is also evidence of developing resistance against imidachloprid and endosulfan,^16^ abamectin,^17^ in some areas of Mexico. To date, there is no report of insecticide resistance in *B. cockerelli* in Oceania.^6^ However, the rapid development of resistance in separate locations suggests that careful resistance management will be needed if resistance is to be avoided in populations in Australia and New Zealand. This will be complex given the need to coordinate strategies across the range of potential host crops.

The use of broad‐spectrum insecticides in controlling *B. cockerelli* has been shown to be toxic to non‐target insects, including potential *B. cockerelli* predators (such as ladybird beetles, lacewings, minute pirate bug, damsel bug) and parasitoid wasps.^6^, ^11^ This can lead to outbreaks of secondary pests such as aphids and mites.^18^, ^19^ Other than secondary pest, resurgence of the *B. cockerelli* population could occur due to loss of predators and parasitoids. Moreover, the excessive use of synthetic insecticides has led to a series of adverse ecological effects, including residue problems, environmental contamination, species displacement and disruption of IPM systems.^16^, ^17^, ^20^, ^21^ Therefore, chemical sprays may not be a long‐term solution for management of *B. cockerelli*.

There is an increasing interest in exploring the potential to control *B. cockerelli* using biological control agents.^22^, ^23^, ^24^ In New Zealand, several insects, spiders and mite species have been found to prey upon *B. cockerelli*.^25^, ^26^, ^27^ Several species of predators are known to prey upon resident species of psyllids, including spiders, beetles (Coleoptera), bugs (Hemiptera) and birds.^28^ However, to date, no report has been published on the biology and efficiency of Australian potential predators of *B. cockerelli*, which are already in the Australian agricultural environment.

Resident natural enemies, especially generalist predators, may play an important role in developing biocontrol strategies against invasive pests.^22^, ^23^, ^24^, ^29^, ^30^ The ladybird beetles (Coleoptera: Coccinellidae) comprises of more than 6000 species and many serve as important biological control agents around the world.^31^ They can feed upon many pests, such as aphids,^32^, ^33^ whiteflies,^34^ mealybugs,^35^ scale insects,^36^ and mites.^37^ The variegated lady beetle, *Hippodamia variegata* Goeze (Coleoptera: Coccinellidae) is considered one of the most important predators for many pests.^38^, ^39^, ^40^, ^41^, ^42^, ^43^, ^44^, ^45^, ^46^, ^47^, ^48^, ^49^ *H. variegata* originated in the Palaearctic region but is now widespread in Australia as well as across the world due to its feeding aggressiveness and high biotic potential.^46^ In Australia, *H. variegata* has been recorded preying on 12 aphid species and one psyllid species in a diversity of crops.^46^ The 11‐spotted ladybird beetle, *Coccinella undecimpunctata* L. (Coleoptera: Coccinellidae), the large spotted ladybird beetle, *Harmonia conformis* Boisduval (Coleoptera: Coccinellidae) and the southern ladybird beetle, *Cleobora mellyi* Mulsant (Coleoptera: Coccinellidae) were reported as a potential biocontrol agent for *B. cockerelli* in New Zealand.^50^, ^51^ In California, Butler and Trumble,^52^ also reported that convergent lady beetle, *Hippodamia convergens* Guérin‐Méneville (Coleoptera: Coccinellidae) was a predator of *B. cockerelli*. In this context, the voracity, food consumption, predation capacity, and reproductive rate might make *H. variegata* a major biocontrol agent for *B. cockerelli* in integrated pest management (IPM) programs.

In this study, we first assessed the demographical parameters of *H. variegata* on *B. cockerelli*, which were compared to those reared on the long‐term resident pest *Myzus persicae* Sulzer (Hemiptera: Aphididae) or on a mixed population of *B. cockerelli* and *M. persicae*. Then, the predation rates (and hence value as a biological control agent) of *H. variegata* on *B. cockerelli* was evaluated through a series of laboratory and greenhouse experiments.

The functional response of *H. variegata* larvae and adults was assessed on different densities of *B. cockerelli* nymphs. Functional response is an important characteristic, which describes the prey consumption by the predator and is a primary means of estimating the pest suppression ability of biocontrol agents.^53^, ^54^, ^55^, ^56^ Therefore, the performance of *H. variegata* in the control of *B. cockerelli* was examined under greenhouse conditions using tomato plants artificially infested with *B. cockerelli*.

## 2 MATERIALS AND METHODS

### 2.1 Ethics statement

As *B. cockerelli* is a listed quarantine species, authorization was obtained from the State Government of Western Australia to maintain colonies and conduct research on the species. All work was conducted in a domestic quarantine greenhouse at Murdoch University, Perth. No special permissions were required for the field collection of insects. None of the species used in this study are endangered or protected.

### 2.2 Insect rearing

#### 2.2.1 Tomato potato psyllid cultures

A colony of *B. cockerelli* was established from individuals originally collected from a capsicum field north of Perth, Western Australia (31.65° S, 115.93° E) in 2019. The stock colony was reared on tomato plants (*Solanum lycopersicum* L.) grown in one‐liter pots within BugDorm‐4S4590 insect rearing cages (47.5 cm × 47.5 cm × 93.0 cm) (MegaView Science Co., Ltd., Taiwan). Wild *B. cockerelli* were introduced frequently to strengthen the culture. The insect rearing cages were maintained in a quarantine greenhouse facility at Murdoch University, Perth, Western Australia. Senesced tomato plants were replaced with fresh ones as required. The insect culture was maintained for six to seven generations under natural light and humidity conditions (average temperature 19.1 °C (Highest temperature ever observed, 31.6 °C), relative humidity (RH) 52.8% and 14 L: 10 D photoperiod (Bureau of Meteorology, Australian Government, site: http://www.bom.gov.au/)) through the experiment period (November 2020 to February 2021).

#### 2.2.2 Green peach aphid cultures

*M. persicae* was collected from a canola field managed by Department of Primary Industries and Regional Development (DPIRD), Northam, Western Australia (31.65° S, 116.69° E) in 2020. A culture was maintained on canola plants (*Brassica napus* L.) grown in 1 L pots within cages. Colonies were maintained for 10–12 generations in the same greenhouse and under the same environmental conditions as *B. cockerelli* (November 2020 to February 2021).

#### 2.2.3 Ladybird beetle cultures

A colony of *H. variegata* was established from individuals collected from a canola field managed by DPIRD in Northam, Western Australia (31.65^°^ S, 116.69° E) in 2020. *H. variegata* was laboratory reared for two to three generations prior to use. Adults and larvae were maintained in plastic boxes (12 cm in diameter and 8 cm in height) with a 4‐cm hole in the lid for ventilation which was covered. Every 5 days, eggs were separated from the culture and transferred to Petri dishes (5 cm × 1 cm) and placed on water‐soaked tissue paper. As they emerged, first‐instar larvae were collected and placed into a new plastic box. The population of *H. variegata* (adults and larvae) were then cultured on an ample supply of a mixed population of *B. cockerelli* and *M. persicae*. The plastic boxes containing ladybirds were kept in the quarantine greenhouse facility at Murdoch University, Perth, Western Australia. The culture was maintained under the same conditions as the other species.

### 2.3 Comparative life table study

**Figure 1:**
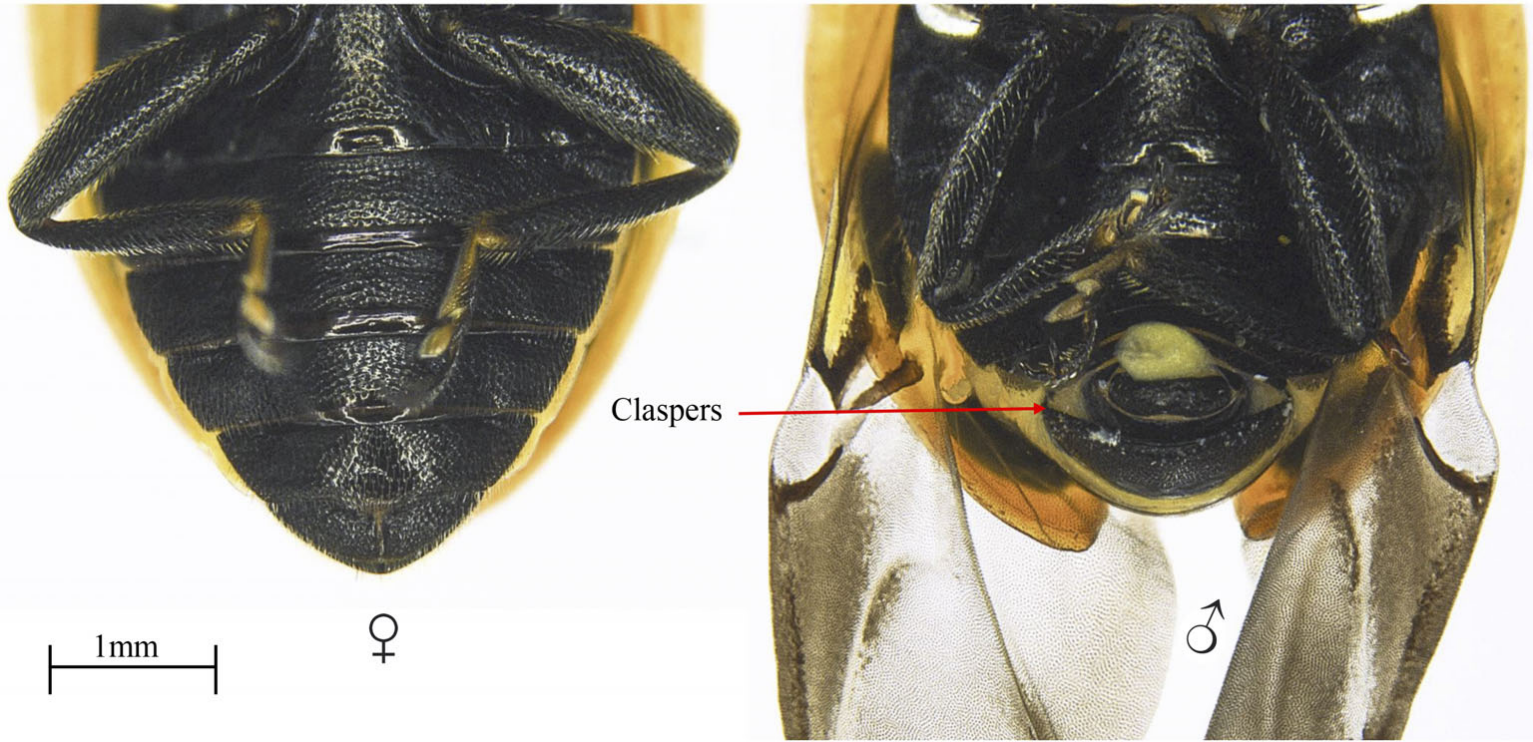
Abdominal section of female (left) and male (right) *Hippodamia variegata*. Males have claspers on the terminal abdominal segment.

To derive the parameters of the life table, 100 eggs of *H. variegata* were collected randomly from the colony and placed on moist filter paper in a Petri dish. The eggs were monitored daily and after 3 days, 45 first instar larvae were transferred individually in a Petri dish (5 cm × 1 cm). Water‐saturated filter paper was placed on the bottom of the Petri dishes to prevent the leaf disks from desiccation during the experimental period. A tomato leaf disk (4.5 cm diameter) was centered upside down on the water‐saturated filter paper. A 2‐cm hole was punched on the lid of Petri dish and covered with fine mesh for ventilation. *H. variegata* larvae initially fed on second instar nymphs of *M. persicae* and third instar nymphs of *B. cockerelli* at the respective prey treatments (*B. cockerelli*, *B. cockerelli* & *M. persicae* or *M. persicae*) and the subsequent instars fed on third instar nymphs of *M. persicae* and fourth instar nymphs of *B. cockerelli*. Prey was provided *ad libitum*. *H. variegata* were observed daily to record survival, development time, pupation time and adult emergence. Each larva was considered an individual replicate for each treatment. After adult emergence, ladybirds were inspected under a hand magnifier to determine their sex (Fig. 1) and pairs that had emerged on the same day were reared together in a plastic container (12 cm in diameter and 8 cm in height) with a 4‐cm hole punched in the lid and covered with fine mesh for ventilation. A mixed population of all prey treatments (*ad libitum*) and a freshly excised tomato shoot were provided in each container every 2 days. The reproductive duration, fecundity of every female and longevity of each female and male were monitored every 2 days until death. The developmental period, fecundity, adult preoviposition period (APOP), and total preoviposition period (TPOP) were evaluated using the experimental data based on the two‐sex age‐stage life table. The age‐specific survival rate and life expectancy were also calculated. The temperature and relative humidity (RH) in the greenhouse were recorded throughout the experiment using a digital data logger. The average temperature and RH was 18.8 °C (ranged from 11.0–31.6 °C) and 55.5% (ranged from 20.8–90.6%), respectively.

### 2.4 Response to prey density

Prey density‐dependent feeding activity was examined for larval and adult *H. variegata* when fed late nymphal instars of *B. cockerelli*. The average temperature and relative humidity (RH) were 19.6 °C (ranged from 15.0–25.7 °C) and 48.9% (ranged from 28.3–84.5%), respectively during the experimental period (17 to 29 December 2020). *H. variegata* larvae and adult (1 day old in terms of emerging into that stage) which had been starved for 24 h prior to the experiment were used. The experiment was conducted in 5‐cm diameter Petri dishes. Water‐saturated filter paper was placed at the bottom of the dish and a tomato leaf disk (4 cm diameter) was placed upside down on the filter paper. Different prey densities (2, 4, 8, 16, 32 and 64 nymphs) were carefully transferred onto each Petri dish using a fine brush. The predators were added to the experimental arenas 1 hour later and then the dish was sealed with parafilm around the edge. The number of *B. cockerelli* nymphs consumed was recorded after 24 h. The bioassay was replicated 10 times for each density.

### 2.5 Greenhouse evaluation

The greenhouse experiment for controlling *B. cockerelli* was conducted using tomato (*S. lycopersicum*) plants from November to December in 2020 in the quarantine greenhouse facility at Murdoch University, Perth, Western Australia. Firstly, 1.5 L plastic pots were filled with potting mix and pasteurized. One‐week old tomato seedlings were then transplanted into the pots and each pot transferred into a cage (93.0 cm × 47.5 cm × 47.5 cm) made of fine mesh. The cages were equipped with a zipper for access.

To ensure that the plants were free of other insects, cages were inspected at the beginning of the experiment. On 22 November 2020, forty adult *B. cockerelli* (approximately 60% females to maintain higher reproduction with lower initial release) were added to each cage. The population of *B. cockerelli* was composed of all life stages (approximately 60% nymphs) at the time of the initial release. After 10 days, first instar (24 h old) *H. variegata* larvae were released on the leaf surfaces of the caged plants at different densities (0, 2, 4, 8 or 16 larvae per plant). There were five replications for each treatment. Each plant served as a replicate. The nymphal stages of *B. cockerelli* predominantly stay on the underside of the leaves and adult *B. cockerelli* also prefer leaf rather than other plant parts. Therefore, Pest populations were monitored on six leaves per plant (two each from the top, middle and bottom thirds of each plant to standardize the insect population from different leaves^57^) by non‐destructive sampling before release of the predators and then every 5 days. The average number of individuals per leaf was determined. Fully senesced leaves were removed from the bottom of the plants so that they did not obstruct watering. They were retained within the cage in case there were still eggs or nymphs on them.

*B. cockerelli* is a phloem feeding insect and causes psyllid yellow diseases (yellowing of young leaves^6^). Therefore, chlorophyll status of the tomato plants was assessed (first sampling date after the release predators and then every 7 days) with a chlorophyll meter (CCM‐200 Plus GPS Chlorophyll Content Meter, Opti‐Sciences, Inc., Hudson, USA). One leaf was sampled from each of the three vertical strata. Chlorophyll status was measured at proximal, middle and distal positions of the leaf to capture any possible variation and the average value determined. At the end of the experiment, the above ground parts of the plants were cut and then dried in an oven at 60 °C for 48 to 72 h and weighed. During this experiment, the temperature ranged from 11 °C to 26 °C and the RH ranged from 29% to 85%.

### 2.6 Statistical analyses

The life table parameters (developmental durations, survival rates, daily fecundities and their demographical parameters) of all *H. variegata* individuals were calculated based on the age‐stage, two‐sex life table with the TWOSEX‐MSChart program.^58^, ^59^, ^60^ Following the method of Chi and Liu,^58^ the survival rate (*s*_*xj*_) (the probability of an individual of age *x* and stage *j* surviving to age *x*_*j*_ and stage *j*) and daily fecundity *f*_*xj*_ (the daily number of eggs laid by an individual at age *x* and stage *j*) were calculated. The age‐specific survival rate (*l*_*x*_) was calculated where, the number of individuals surviving to age x where *m* is the number of stages:

$$l_{x}=\sum_{j=1}^{m}s_{\mathrm{xj}}$$

The age‐specific fecundity (*m*_*x*_) expressed as the number of female offspring per female of age *x* where *j* is the number of stages was calculated as follows:

$$m_{x}=\frac{\sum_{j=1}^{m}s_{\mathrm{xj}}f_{\mathrm{xj}}}{\sum_{j=1}^{m}s_{\mathrm{xj}}}$$

The net reproductive rate (*R*_*0*_) is the degree to which a population will increase after one generation, and was calculated as follows:

$$R_{0}=\sum_{x=0}^{\infty }l_{x}m_{x}$$

The Euleri‐Lotka formula was followed to estimate the intrinsic rate of increase (*r*), which is the maximum exponential multiplication rate of the whole population with the age indexed from zero.^61^ It was calculated as follows:

$$\sum_{x=0}^{\infty }e^{-r\left(x+1\right)}l_{x}m_{x}=1$$

The finite rate of population growth (*λ*) is the rate as the time approaches infinity and the population reaches the stable age‐stage distribution was calculated as follows:

$$\lambda =e^{r}$$

The mean generation time (*T*), the time between oviposition in one generation and in the following generation, was calculated as follows:

$$T=\frac{\ln R_{0}}{r}$$

The age‐stage‐specific life expectancy (*e*_*xj*_), where *s*'_*iy*_ represents the probability that an individual at age *x* and stage *j* will survive to age *i* and stage *y* was calculated according to the method described by Chi and Su,^62^ as follows:

$$e_{\mathrm{xj}}=\sum_{i=x}^{\infty }\sum_{y=j}^{m}s{´}_{\mathrm{iy}}$$

The reproductive value (*v*_*xj*_) is defined as the contribution of individuals at age *i* and stage *y* to the future population.^63^ It was calculated as follows by the method described by Tuan *et al*.,^64^:

$$v_{\mathrm{xj}}=\frac{e^{r\left(x+1\right)}}{s_{\mathrm{xj}}}\sum_{i=x}^{\infty }e^{-r\left(i+1\right)}\sum_{y=j}^{m}s{´}_{\mathrm{iy}}f_{\mathrm{iy}}$$

Bootstrap technique with 100 000 replications were used to calculate the mean and standard error of the population parameters to minimize the variability in the results.^65^ Differences between treatments and control were compared by performing an analysis of variance (ANOVA) test.

Functional response can be described by Holling type II functional response model.^66^ The parameters are calculated as the equation using the software SPSS 24.0:

$$N_{a}=a^{´}\mathrm{TN}/\left(1+a^{´}T_{h}N\right)$$

Where, *N*_*a*_ is the net prey number attacked by a predator per time unit (here, time (*T*) is 24 h = 1 day); *a*' is the rate of successful attack by a single predator; *N* is the initial prey density; *T* is the exposure time to the predator (1 day); *T*_h_ is the duration of one prey consumption by predator (handling time). Data on the number of psyllids were first subjected to a square root transformation to correct for homogeneity of variances.

The mean number of adult and nymphal *B. cockerelli* were compared for the different treatments to evaluate the efficacy of the predator for control in the greenhouse experiment. The differences between the different treatments and controls were examined by performing an ANOVA test. The means of different treatments were subjected to a *post hoc* Tukey's test (*P* < 0.05). The proportional suppression (*D*) of *B. cockerelli* at the final sampling date (28 December 2020) were calculated compared to untreated control as follows:

$$D=\left(d_{\text{T0}}-d_{\mathrm{Ti}}\right)/\left(d_{\text{T0}}\right)\times 100\%$$

Where, *d*_T0_ and *d*_Ti_ were the mean psyllid densities (number of *B. cockerelli* per leaf) in the untreated controls and the treatments. One‐way ANOVA followed by an HSD Tukey test was applied to compare SPAD and dry weight data using SPSS 24.0, SPSS Inc, Chicago, IL.^67^

## 3 RESULTS

### 3.1 Life table and population parameters

The duration of the total immature period differed significantly (*P* < 0.05). *H. variegata* that fed on *M. persicae* exhibited the shortest pre‐adult period (18.07 ± 0.24 days). Adult longevity (both female and male) did not differ significantly in all treatments (Table 1). All *H. variegata* eggs hatched at the same time (3 days). The duration of the first instar larval stage was significantly shorter in *H. variegata* that fed on *M. persicae* compared to the other two treatments (Table 1). The duration of the third instar larval stage also differed between treatments (*M. persicae* > *B. cockerelli* > *B. cockerelli* & *M. persicae*). There was no significant difference in the duration of the second and fourth instar larval stages for insects reared on the different diets (Table 1). The longest total pupal period (5.69 ± 0.13 Days) was obtained from the beetles which fed on the mixed population of *B. cockerelli* & *M. persicae*.

**Table 1:** Developmental time, adult longevity and reproduction parameters of *Hippodamia variegata* when raised on different diets: *Bactericera cockerelli*, *Myzus persicae* or a combination of the two (mean ± SE)

Stage	Developmental time (d) (mean ± SE)						*df*	*F*	*P*
	*n* (*N*)	*B. cockerelli*	*n* (*N*)	*B. cockerelli* & *M. persicae*	*n* (*N*)	*M. persicae*			
Egg	15(15)	3 ± 0a	15(15)	3 ± 0a	15(15)	3 ± 0a	2,42		
L1	14(15)	3.43 ± 0.13a	15(15)	3.33 ± 0.15a	14(15)	2.71 ± 0.12b	2,40	7.3	0.002
L2	14(14)	2.50 ± 0.13a	15(15)	2.60 ± 0.13a	14(14)	2.21 ± 0.11a	2,40	2.43	0.10
L3	14(14)	2.21 ± 0.11ab	15(15)	2.13 ± 0.09b	14(14)	2.57 ± 0.13a	2,40	4.13	0.02
L4	14(14)	2.93 ± 0.33a	14(15)	2.50 ± 0.17a	14(14)	2.79 ± 0.15a	2,40	0.75	0.48
Pupa	13(14)	4.85 ± 0.18b	13(14)	5.69 ± 0.13a	14(14)	4.79 ± 0.23b	2,37	6.67	0.003
Pre‐adult duration	13(13)	19.00 ± 0.40ab	13(13)	19.31 ± 0.25a	14(14)	18.07 ± 0.24b	2,37	4.37	0.02
Adult longevity									
Female	8	108.0 ± 2.8a	7	103.6 ± 6.5a	9	100.4 ± 3.0a	2,21	0.89	0.43
Male	5	102.8 ± 4.9a	6	96.2 ± 5.7a	5	106.6 ± 2.5a	2,13	1.27	0.31
Reproduction parameter									
APOP	8	1.88 ± 0.39a	7	1.43 ± 0.29a	9	1.56 ± 0.29a	2,21	0.45	0.64
TPOP	8	21.13 ± 0.50a	7	20.57 ± 0.42a	9	19.67 ± 0.54a	2,21	2.18	0.14
Oviposition period	8	36.00 ± 1.20a	7	36.86 ± 2.95a	9	35.11 ± 2.46a	2,21	0.14	0.87
Total fecundity (Eggs/female)	8	766.8 ± 55.13b	7	1238.6 ± 103.4a	9	1270.3 ± 132.0a	2,21	7.2	0.004

*Note:* N, total replicate number; n, effective replicate number; Values followed by the different lowercase letters within a row are significantly different using ANOVA (*P* < 0.05). APOP is the adult pre‐oviposition period (from emergence to first oviposition); TPOP is the total pre‐oviposition period (from hatching to first oviposition). Standard errors (SE) were estimated by bootstrapping (100 000 replications).

Fewer eggs were produced (*P* < 0.05) when feeding on *B. cockerelli* than the other two diets. However, there was no difference also among the diets in terms of adult pre‐oviposition period (APOP), the total pre‐oviposition period (TPOP) and oviposition period (Table 1).

**Figure 2:**
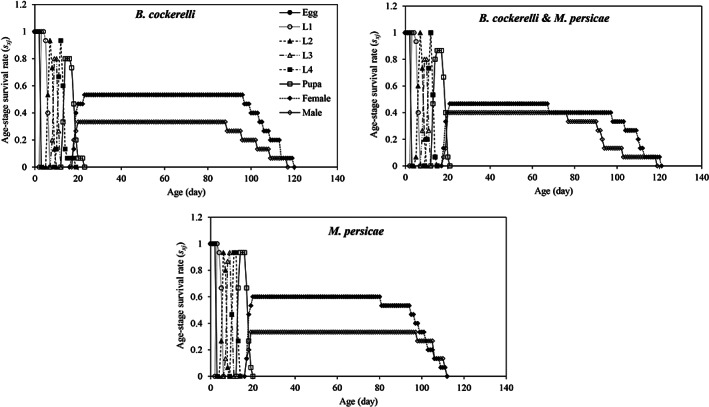
Age‐stage survival rate (*s*_*xj*_) of *Hippodamia variegata* in different treatments.

**Figure 3:**
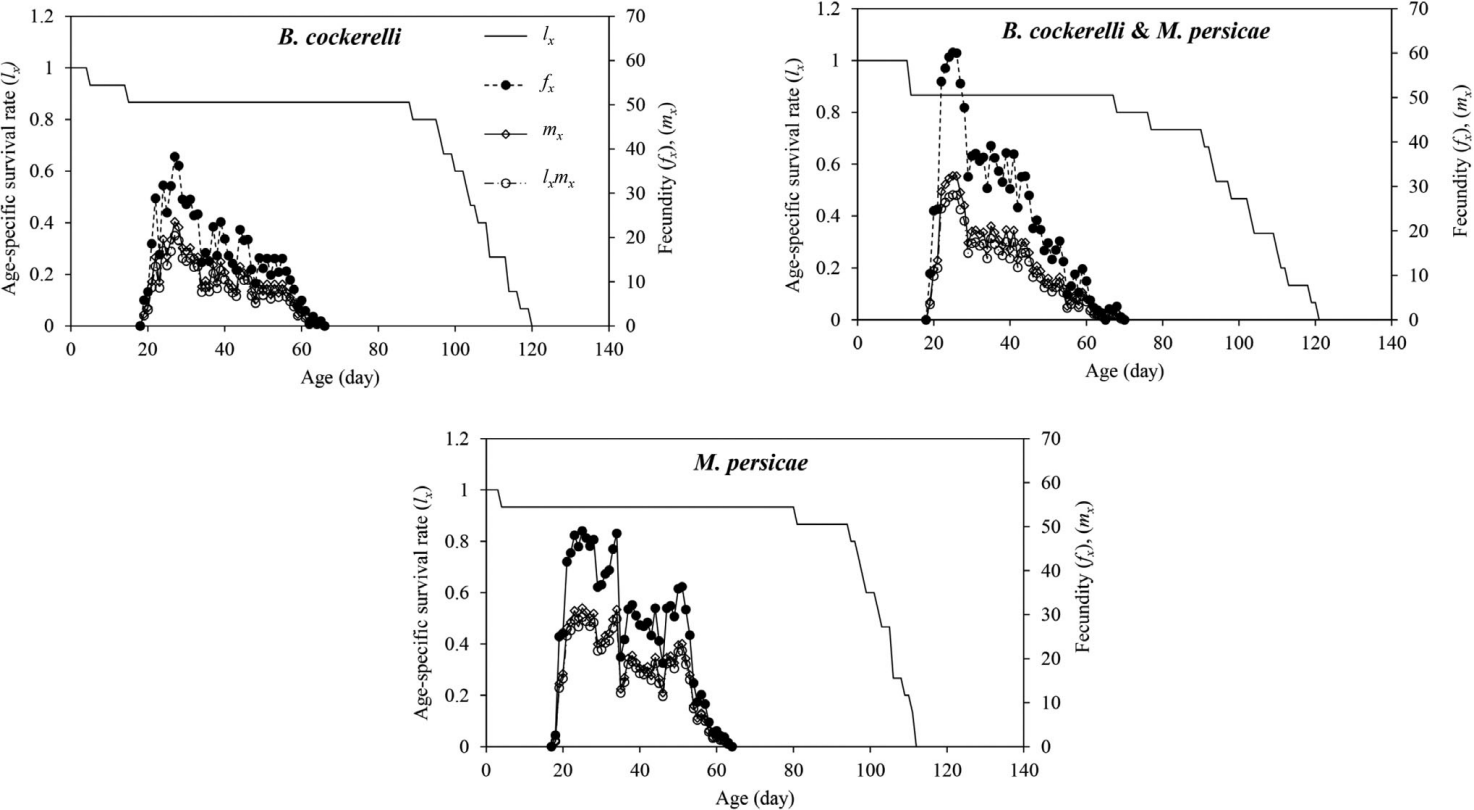
Age‐specific survival rate (*l*_*x*_), female fecundity (*f*_*x*_), fecundities (*m*_*x*_) and age‐specific net maternity (*l*_*x*_ *m*_*x*_) of *Hippodamia variegata* in different treatments.

The adult female age‐stage‐specific survival rate (described by the *s*_*xj*_ curve) was high when reared on *B. cockerelli* (0.53 for adult female) or *M. persicae* (0.60 for adult female) but slightly lower when reared on the mixed population of *B. cockerelli* & *M. persicae* (0.47 for adult female) (Fig. 2). The age‐stage survival rate (*l*_*x*_) curve includes all individuals of the cohort and is a simplified version of the *s*_*xj*_ curves (Fig. 3). The *l*_*x*_, *f*_*x*_, and *m*_*x*_ curves showed that both the survival rate and fecundity were lowest when *H. variegata* was reared on *B. cockerelli*. The approximate periodic peaks in reproduction are represent by the *m*_*x*_ curve. The highest age‐specific fecundity, *m*_*x*_ (32.38 eggs) and age stage‐specific fecundity, *f*_*x*_ (60.14 eggs) values derived from the TWOSEX‐MSChart program were observed at 25 days post‐hatching for *H. variegata* reared on the mixed population of *B. cockerelli* & *M. persicae*. When *H. variegata* fed on *B. cockerelli* or *M. persicae* only, the reproductive peak occurred later; at age 27 days (*m*_*x*_ = 23.54; *f*_*x* =_ 38.25) and 34 days (*m*_*x*_ = 31.14; *f*_*x* =_ 48.44), respectively.

**Figure 4:**
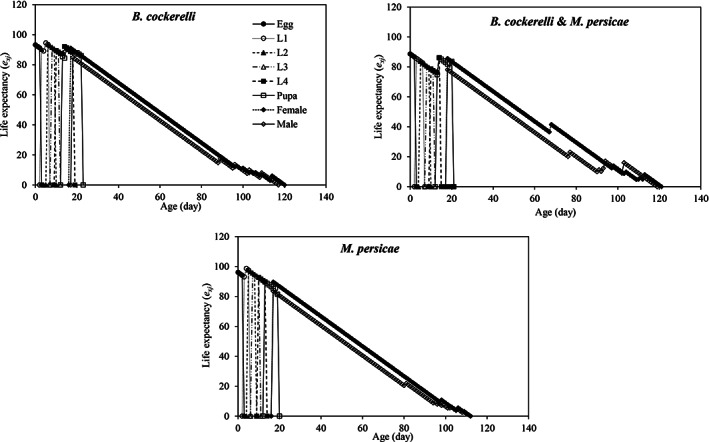
Age‐stage‐specific life expectancy (*e*_*xj*_) of *Hippodamia variegata* in different treatments.

The age‐stage life expectancy (*e*_*xj*_) of the newly hatched larvae of *H. variegata* were 90.2 (on *B. cockerelli*), 85.67 (on a mixed population of *B. cockerelli* & *M. persicae*), and 93.07 (on *M. persicae*) (Fig. 4). However, the life expectancy of the adult *H. variegata* was shorter when feeding on *M. persicae* than the other diets. Regardless of the prey species, adult female life expectancies were generally greater than those of adult males.

**Figure 5:**
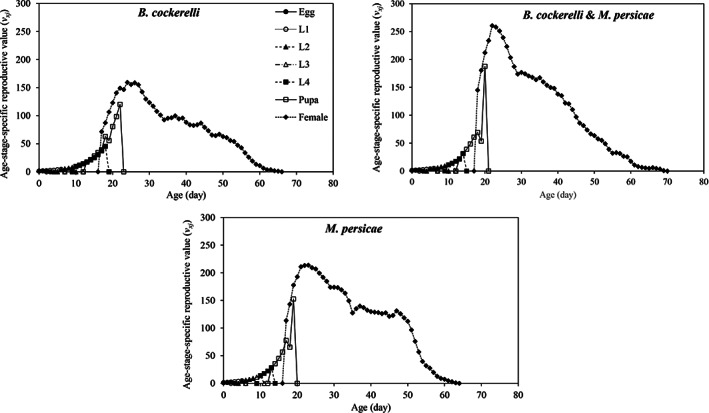
Age‐stage‐specific reproductive value (*v*_*xj*_) of *Hippodamia variegata* in different treatments.

The age‐stage‐specific reproductive values (*v*_*xj*_) (Fig. 5). The *v*_*xj*_ significantly increased when *H. variegata* began to produce eggs. The highest *v*_*xj*_ of the adult females was observed at 22 days (260.67) when reared on the mixed population of *B. cockerelli* & *M. persicae*. In contrast, the peak reproductive value also occurred at 22 days (213.22) when feeding on *M. persicae* while for prey species *B. cockerelli v*_*xj*_ reached a peak after 24 days (159.25). In all treatments, after reaching to the highest value *v*_*xj*_ declined gradually with the age.

The population parameters, net reproductive rate (*R*_*0*_) and mean generation time (*T*), intrinsic rate of increase (*r*), finite rate of increase (*λ*), of *H. variegata*, reared on *B. cockerelli*, mixed population of *B. cockerelli* & *M. persicae* and *M. persicae* were calculated based on data from the entire cohort (Chi and Liu, 1985) (Table 2). According to statistical analysis, the *R*_*0*_ were not significantly different among all three treatments (Table 2). The mean generation time was significantly decreased in the following order: *B. cockerelli* > mixed population of *B. cockerelli* & *M. persicae* > *M. persicae* (Table 2). However, *r* and *λ* were minimized when fed on *B. cockerelli*.

**Table 2:** Population parameters of *Hippodamia variegata* when raised on different diets: *Bactericera cockerelli*, *Myzus persicae* or a combination of the two (mean ± SE)

Parameters	*B. cockerelli*	*B. cockerelli & M. persicae*	*M. persicae*
Net reproductive rate, *R*_*0*_	408.9 ± 102.3a	578.0 ± 165.5a	762.2 ± 177.0a
Mean generation time, *T* (d)	30.19 ± 0.64a	28.89 ± 0.66ab	28.44 ± 0.42b
Intrinsic rate of increase, *r* (d^−1^)	0.20 ± 0.01b	0.22 ± 0.01a	0.23 ± 0.01a
Finite rate of increase, *λ* (d^−1^)	1.22 ± 0.01b	1.25 ± 0.02a	1.26 ± 0.01a

*Note*: Values followed by the different lowercase letters within a row are significantly different at the 5% confidence level. The standard error (SE) was estimated by bootstrapping (100 000 replications).

### 3.2 Response to prey density

**Figure 6:**
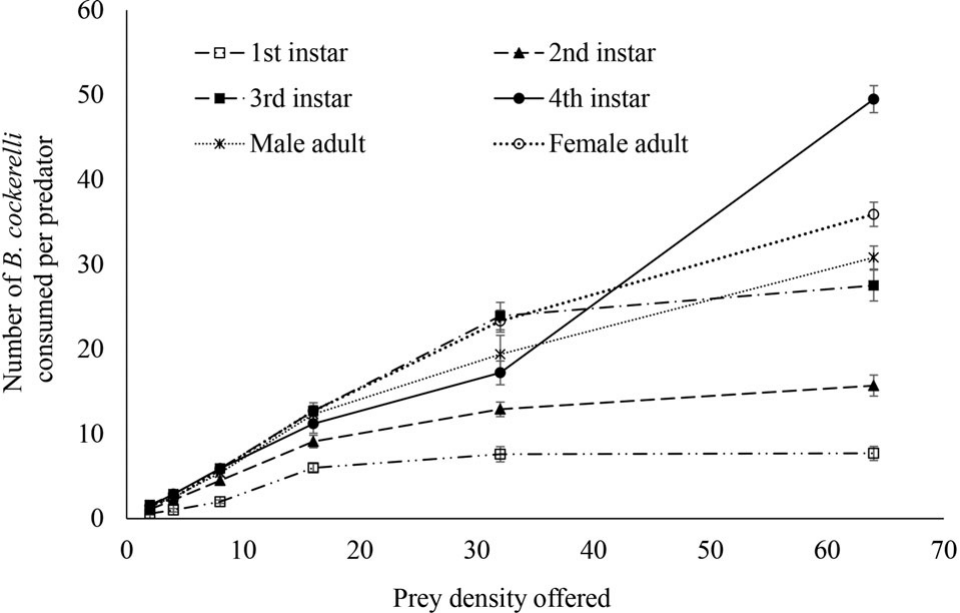
Mean number of *Bactericera cockerelli* nymphs consumed by *Hippodamia variegata* in 24 h at different prey densities (mean ± SE).

The functional response for larval and adult stages of *H. variegata* feeding on different densities of the nymph of *B. cockerelli* were successfully fitted to the Holling II disk equation (Table 3). For each life stage, the predation rate increased with increasing prey density (Table 3 and Fig. 6). The mean numbers of prey consumed by 4th larval instar and adult were substantially higher (*df* = 5.54; *F* = 109.23; *P* < 0.05) than that of males when prey density was between 32 and 64. When offered 16 *B. cockerelli* nymphs, only 11–13 could be consumed by one 4th instar larva or adult ladybird within 24 h. The 4th instar larvae showed minimum handling time compared to other stages of *H. variegata* in this experiment (Table 3).

**Table 3:** Variation in the number of *Bactericera cockerelli* consumed in 1 day (24 h) by *Hippodamia variegata* as influenced by the density of prey presented (as described as the Functional response equation) (mean ± SE)

Treatment	Functional response equations	Attack rate (*a´*)	Handling time (*T*_*h*_, *d*)	*R* ^*2*^ value
1st instar	N_a_ = 0.299 × *N*_*t*_/(1 + 0.173*N*_*t*_)	3.334 ± 0.198	0.052 ± 0.047	0.986
2nd instar	N_a_ = 0.563 × *N*_*t*_/(1 + 0.027*N*_*t*_)	1.775 ± 0.039	0.015 ± 0.009	0.998
3rd instar	N_a_ = 0.803 × *N*_*t*_/(1 + 0.015*N*_*t*_)	1.246 ± 0.034	0.012 ± 0.008	0.997
4th instar	N_a_ = 0.756 × *N*_*t*_/(1 + 0.011*N*_*t*_)	1.323 ± 0.018	0.008 ± 0.004	0.999
Male adult	N_a_ = 0.743 × *N*_*t*_/(1 + 0.020*N*_*t*_)	1.346 ± 0.068	0.015 ± 0.016	0.990
Female adult	N_a_ = 0.730 × *N*_*t*_/(1 + 0.014*N*_*t*_)	1.369 ± 0.089	0.010 ± 0.021	0.984

### 3.3 Greenhouse evaluation

**Figure 7:**
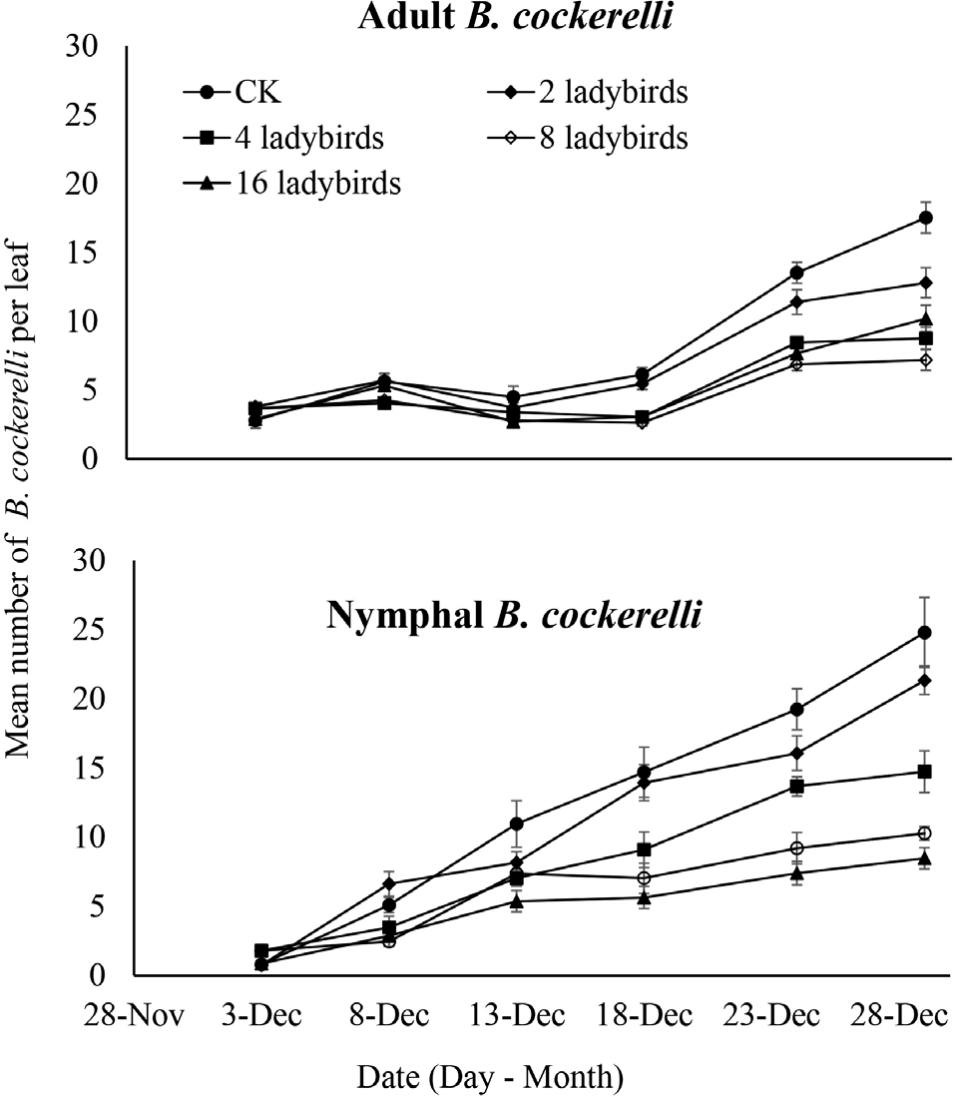
Population fluctuations of adult and nymphal stages of *Bactericera cockerelli* in greenhouse tomato under different treatments of *Hippodamia variegata*.

The increasing densities of *H. variegata* larvae substantially reduced *B. cockerelli* densities by the final sampling date compared to the untreated control treatment (Fig. 7). Population fluctuations in the nymphal and adult *B. cockerelli* were observed in different treatments during each of the experimental periods. At the beginning of the experiment (3 December 2020), the initial densities of nymphal and adult *B. cockerelli* was counted in each of the treatments and did not vary significantly among replicates (larvae: *F* = 4.02; *df* = 4; *P* = 0.15; adult: *F* = 2.27; *df* = 4; *P* = 0.097). This confirmed that the *B. cockerelli* populations were established and distributed evenly on tomato plants. In the untreated control (CK), the populations of nymphal and adult *B. cockerelli* decreased slightly around the third sampling date (as some of initially released adults may die) and then increased until the last sampling date: larvae by two‐fold and adults three‐fold (Fig. 7). In general, the control capacity on both nymphal and adult *B. cockerelli* on greenhouse tomatoes increased in proportion to the initial density of predatory larvae: compared to the untreated control, the density decline of *B. cockerelli* population at the end of the sampling was 14%, 41%, 59%, and 66% for nymph (*F* = 23.32; *df* = 4; *P* < 0.05) and 27%, 50%, 59%, and 42% for adult (*F* = 17.92; *df* = 4; *P* < 0.05), respectively, for initial densities of 2, 4, 8, and 16 *H. variegata* larvae.

**Figure 8:**
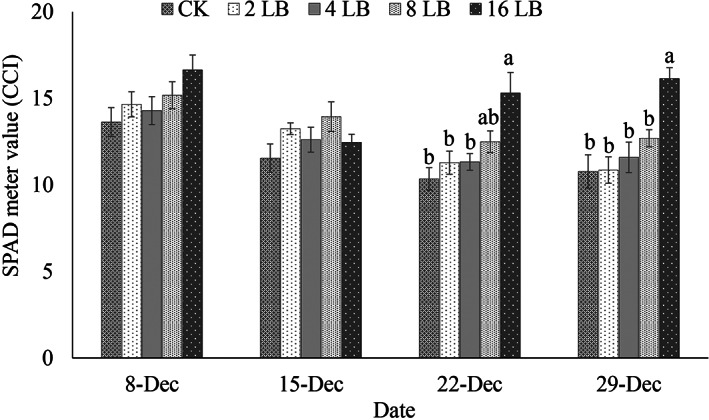
Changes over time in the Soil and Plant Analysis Development (SPAD) readings (chlorophyll measurements) on leaves treated with different densities of *Hippodamia variegata* (mean ± SE). Different letters indicate significant differences at *P* ≤ 0.05. Here, untreated control (CK) and ladybirds (LB).

**Figure 9:**
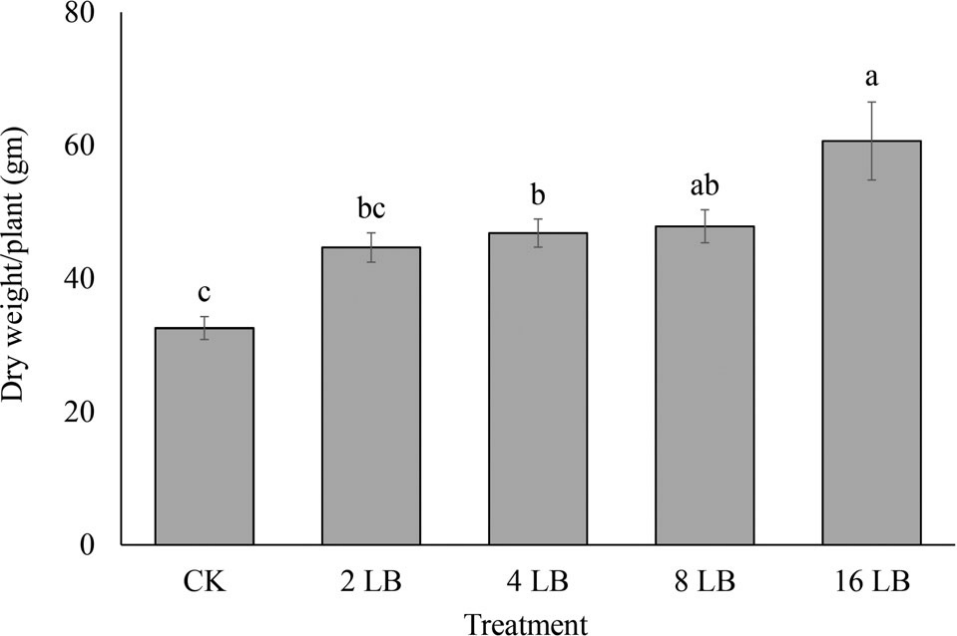
Dry weight of plant (above ground part) treated with different densities of *Hippodamia variegata* (mean ± SE). Different letters indicate significant differences at *P* ≤ 0.05. Here, untreated control (CK) and ladybirds (LB).

In comparison to CK plants, the chlorophyll content of the leaves (as measured using SPAD values) was higher with a higher number of ladybirds (Fig. 8). In the last two sampling dates (8th and 29th December), the SPAD values were significantly higher than the control when either 8 or 16 *H. variegata* was present (*F* = 6.43; *df* = 4; *P* < 0.05; *F* = 8.49; *df* = 4; *P* < 0.05, respectively). The dry weight of above‐ground parts of tomato plants also varied significantly among different treatments (*F* = 9.44; *df* = 4; *P* < 0.05) (Fig. 9). With the releases of *H. variegata* larvae at densities of 2, 4, 8, and 16 per plant, dry weight was increased by 37%, 44%, 47% and 86%, respectively, compared to untreated control. Overall, compared with the untreated control, densities of nymphal and adult *B. cockerelli* were reduced, SPAD value and dry weight were increased.

## 4 DISCUSSION

This is the first report on the potential of a resident predator to contribute to the control of the invasive pest *B. cockerelli* in Australia. The results showed a significant impact of *H. variegata* on the various life stages of the pest and also demonstrated the suitability of the novel prey as a food source for the predator. This study provides basic information required for the potential use of *H. variegata* in biocontrol programs against the invasive pest *B. cockerelli*. It improves our understanding of predatory behavior of *H. variegata* on *B. cockerelli*, and the impact on predator biology. This information will contribute to the development of efficient and environmentally sound pest management strategies for *B. cockerelli*.

*H. variegata* is an aggressive generalist predator within Australian agro‐ecological systems and soon after being recorded in Australia was reported to prey on aphid and psyllid species.^46^ Prey species of *H. variegata* include important agricultural pests in Australia including *Aphis gossypii* Glover (Hemiptera: Aphididae),^68^ *M. persicae* ^69^ and important current quarantine threats such as *Aphis fabae* Scopoli (Hemiptera: Aphididae).^70^ Our results showed that the tomato potato psyllid can also be a suitable diet for supporting the development and reproduction of *H. variegata*. The life table analysis showed that while there were significant differences in growth and reproduction of *H. variegata* reared on *B. cockerelli versus M. persicae*, the impacts were generally small. Therefore, the novel prey, *B. cockerelli* sustained population development of *H. variegata*.

Population growth and the pre‐ovipositional period were similar to those reported by Dehkordi *et al*.,^71^ for *H. variegata* reared in the laboratory, and by Kontodimas and Stathas,^47^ for *H. variegata* grown in outdoor cages. *H. variegata* had a significantly higher rate of population growth (both the intrinsic (*r*) and finite (*λ*) rate of increase) when reared on *M. persicae* than on the other diets, although λ for the *B. cockerelli* diet was only 3% lower with *r* being 13% lower. Thus, while *B. cockerelli* can support the establishment of *H. variegata* populations, the potential rate of population growth is slower.

Predators are expected to have a higher *r* value with a short pre‐ovipositional period if fecundity is similar.^72^ For other species of ladybirds, a higher intrinsic rate of increase was observed with a short pre‐ovipositional period^73^, ^74^ but in those studies, measures of fecundity also varied; population growth being higher with higher fecundity. In the present study, the pre‐ovipositional period did not differ significantly between the diets, population growth rate appearing to be driven primarily by differences in fecundity. While the population growth rate was higher for *H. variegata* feeding on *M. persicae* than on *B. cockerelli*, the net reproductive rate (*R*_*0*_) and mean generation time (*T*) did not vary significantly. Although not significant, the numerical difference in *R*_*0*_ between the diets was large, with non‐significance being due to the high variability. The proportional difference in *R*_*0*_ between the *B. cockerelli* and *M. persicae* diets was similar to the difference in fecundity, with *R*_*0*_ for the *B. cockerelli* diet being 54% that of the *M. persica*e diet and fecundity being 60% that of *M. persicae* diet. This is consistent with the link between the parameters proposed by Chi and Su.^62^

There was very little difference in the development of the predator when reared on the various diets: the rates of pre‐adult development where generally not different, nor were there significant differences in the pre‐ovipositional periods (APOP and TPOP) or the longevity of the male or female adults. This might suggest that the prey provide diets of similar nutritional value.^75^ However, this contrasts dramatically to the marked difference seen in fecundity of the predator when reared on the different prey and the resultant differences in population growth parameters (*r* and *λ*). The contrast may relate to differences in the dietary requirements of the predator for development and for reproduction. Omkar and Srivastava^76^ fed *Coccinella septempunctata* L. (Coleoptera: Coccinellidae) on six different species of aphids. Their data showed a close correlation between the impact of the prey species on the predator's fecundity on the one hand and measures of its growth or development on the other. However, the results of Bonte *et al*.,^77^ for *Adalia bipunctata* L. (Coleoptera: Coccinellidae) reared on various artificial and factitious diets show that such a correlation does not always hold. Indeed, the results of Ali *et al*.,^78^ with the coccinellids, *Propylea japonica* Thunberg (Coleoptera: Coccinellidae) and *Harmonia axyridis* Pallas (Coleoptera: Coccinellidae) show that a diet may be adequate for supporting growth and development but not enable any egg production. Fecundity of ladybirds has previously been shown to be influenced by the size and moisture content of the prey species.^79^, ^80^, ^81^ As *B. cockerelli* is smaller and has a harder body than *M. persicae*, it may contribute to a poorer quality diet as reflected in fecundity and *R*_*0*_. All of our studies were conducted under greenhouse conditions. It is known that in the field coccinellids, like other beetles, augment their diet with other material such as pollen.^82^ This may alter the impact of the *B. cockerelli* diet on predator fecundity.

The arena studies showed that all larval stages and adults of *H. variegata* consumed *B. cockerelli* nymphs. The data fitted a type II functional response to prey density as seen in other coccinellids.^83^, ^84^, ^85^, ^86^ That is, as the density of prey increased, a decreasing proportion of prey was consumed until a plateau consumption rate was reached. However, the attack rate (*a*') was higher and the handling time (*T*_*h*_) shorter than reported for *H. variegata* attacking other prey species.^83^, ^86^, ^87^, ^88^ The fourth instar larvae had a higher daily consumption of *B. cockerelli* nymph than the other juvenile stages or the adult forms. This is reflected particularly in the short handling time. The high consumption of the fourth instar relative to the other larval stages, corresponds to the results of Jafari and Goldasteh^88^ and Farhadi *et al*.,^83^ for *H. variegata* on *A. fabae*. Similarly, Lee and Kang^89^ found that the fourth instar of the coccinellid *H. axyridis* had the highest consumption rate on *A. gossypii*. While the attack rate of the first instar larvae was high in our study (perhaps reflecting a faster movement rate of first instar of *H. variegata*, as they are smaller than their later stages), the handling time of the prey was much longer, resulting in a low daily consumption. Body size of a predator influence its nutritional demand and mobility influences predation capacity, both of which may influence their efficiency as a control agent.^90^ The low potential consumption rate of the early instars and very high rates for the third and fourth instars will need to be taken in to account in defining appropriate release strategies or management strategies for the predator.

The significant suppression of the *B. cockerelli* population growth in greenhouse tomato demonstrated the potential of *H. variegata* for the management of pest populations, particularly in the greenhouse environment. Densities of 8 and 16 *H. variegata* larvae per plant resulted in the same level of suppression for the given level of *B. cockerelli* infestation. In laboratory and greenhouse studies, O'Connell *et al*.,^91^ and Pugh *et al*.,^51^ demonstrated that the southern ladybird, *C. mellyi* could also suppressed *B. cockerelli* on potato plants.

The lower *B. cockerelli* numbers on tomato plants with the predator present, was associated with higher chlorophyll content in the last two dates of sampling (22 December and 29 December). In turn, this was associated with higher plant dry matter. Lower photosynthetic rates and premature decline and death of plants have been reported due to CLso transmitted by *B. cockerelli*.^92^, ^93^ However, this pathogen is not yet present in Australia.^5^ The lower chlorophyll and dry matter of plants with higher *B. cockerelli* numbers in our study was presumably a direct impact of psyllid feeding. However, the transmission of other pathogens that might be associated with the psyllid cannot be ruled out.^94^, ^95^, ^96^ Thus, the presence of the *H. variegata* resulted in suppression of *B. cockerelli* numbers and improved plant growth. Broadly, the outcome of our studies described above suggested there may be potential for *H. variegata* to impact the development of *B. cockerelli* populations. However, the current work was conducted in no‐choice conditions. On‐going experimentation is exploring prey preference and the impact of habituation on the predator's prey selection which will be important in industry situations where alternative prey is available.

## 5 CONCLUSIONS

This study is the first investigation exploring the life history and voracity of *H. variegata* as a predator of *B. cockerelli* and its potential as a biocontrol agent to manage the invasive pest in Western Australia. Our results showed that *H. variegata* can successfully suppress *B. cockerelli* populations in greenhouse conditions. Further, the predator can survive and reproduce when feeding on *B. cockerelli* but the population growth rate may be somewhat lower. The greenhouse trial showed that *H. variegata* has potential as a biocontrol agent for controlling invasive *B. cockerelli* in IPM programs, which involves augmentative releases of *H. variegata* in an IPM program, along with the conservation and enhancement of naturally occurring populations of this predator in Solanaceae crop systems in Australia.

## CONFLICT OF INTEREST

The authors have no conflicts of interest to declare that are relevant to the content of this article.

## Data Availability

The data that support the findings of this study are available on request from the corresponding author. The data are not publicly available due to privacy or ethical restrictions.
